# No relationship between early postnatal testosterone concentrations and autistic traits in 18 to 30-month-old children

**DOI:** 10.1186/s13229-016-0078-8

**Published:** 2016-02-17

**Authors:** Karson T. F. Kung, Mihaela Constantinescu, Wendy V. Browne, Rebecca M. Noorderhaven, Melissa Hines

**Affiliations:** Department of Psychology, University of Cambridge, Free School Lane, Cambridge, CB2 3RQ UK

**Keywords:** Autism, Testosterone, Sex differences, Gender differences, Postnatal development

## Abstract

**Background:**

Some previous research has suggested that testosterone prenatally contributes to gender differences in autistic traits, but little is known about the role of testosterone during early postnatal development (mini-puberty). Two prior studies found no sex difference in testosterone postnatally in saliva samples and detected little to no relationship between testosterone postnatally and autistic traits in toddlers. These findings may reflect late measurements of testosterone at 3 to 4 months of age, after the peak of mini-puberty at 1 to 3 months of age. The present study examined the relationship between testosterone at 1 to 3 months of age and autistic traits at 18 to 30 months of age.

**Findings:**

Testosterone was measured in saliva samples collected from children at 1 to 3 months of age. When the children (40 boys, 47 girls) reached 18 to 30 months of age, parents completed the Quantitative Checklist for Autism in Toddlers (Q-CHAT). Boys had higher concentrations of testosterone postnatally and higher Q-CHAT scores than girls. However, testosterone did not correlate with Q-CHAT scores in boys, girls, or the entire sample.

**Conclusions:**

The current results suggest that testosterone during the early postnatal period does not contribute to later autistic traits. Given our relatively small samples and therefore limited power, however, further research could usefully examine if testosterone in saliva samples collected during the peak of mini-puberty in larger groups predicts autistic traits or other traits that show gender differences.

## Findings

### Background

One of the most consistent findings in autism spectrum conditions (ASCs) research is that males are more likely than females to be diagnosed with the conditions [1]. In addition, in the general population, males on average score higher on measures of autistic traits than females [2–5].

Studying the causal mechanisms involved in this gender bias may provide insights into the aetiology of ASCs. It has been proposed that ASCs reflect an ‘extreme male brain (EMB)’ and that androgens during early development are a key neurobiological factor underlying development of an EMB [6, 7]. Some prior studies have found that testosterone prenatally is significantly related to subsequent autistic traits [8–11]. A recent study compared 128 males with an ASC diagnosis to 217 matched controls without an ASC diagnosis and found no significant difference in testosterone that had been measured in their amniotic fluid [12]. That same study, however, found that the ASC group scored significantly higher than the controls on a latent steroidogenic factor that was derived from measures of five hormones (progesterone, 17α-hydroxy-progesterone, androstenedione, testosterone, and cortisol) assayed in amniotic fluid [12].

Little is known about whether androgen exposure during early infancy relates to later autistic traits. There is a transient surge of sex steroids, called ‘mini-puberty’ , in male infants, with testosterone concentrations peaking at about 1 to 3 months postnatal, before declining to low childhood values by around 6 months of age [13, 14]. Testosterone during mini-puberty has been shown to influence development of the male genitalia and reproductive function [15] and has been associated with postnatal penile and testicular growth in healthy infants [16]. Since brain plasticity remains high throughout the early postnatal period [17], it is possible that mini-puberty contributes to neurobehavioral sexual differentiation, as well.

Two studies have examined the influences of testosterone during mini-puberty on autistic traits. These studies related testosterone assayed from saliva samples collected at 3 to 4 months of age to parent-reported autistic traits during toddlerhood. The first study found no correlation between testosterone and scores on the Quantitative Checklist for Autism in Toddlers (Q-CHAT) in boys (*n* = 15), girls (*n* = 20), or the entire sample at age 18 to 35 months [8]. The second study reported a significant positive correlation between testosterone and scores on a subscale of the Brief Infant–Toddler Social and Emotional Assessment (BITSEA) in 47 boys and 37 girls combined at age 18 to 24 months but did not report any within-sex correlation [18]. Notably, neither study found a sex difference in testosterone concentrations, perhaps because saliva samples were collected after the peak of mini-puberty. One of the studies also found no gender difference in autistic traits [18]. The lack of differences between boys and girls limits the studies’ implications for the neurobehavioral effects of mini-puberty on autistic traits.

The present study explored the relationship between salivary testosterone during the peak of mini-puberty, at 1 to 3 months of age, and parent-reported autistic traits, as assessed by the Q-CHAT, at 18 to 30 months of age. Saliva sampling was employed since it affords a non-invasive approach. The Q-CHAT was used because prior studies have found gender differences in Q-CHAT scores [2, 8].

### Methods

#### Participants and design

Participants were recruited from a larger longitudinal study investigating the influences of testosterone during mini-puberty on child development. The pattern of the postnatal surge appears to differ for full term (37 or more weeks of gestation) and preterm infants [16], so only full term infants were recruited. Parents of 87 (47 girls) healthy, full term infants were recruited in Cambridgeshire, England, and saliva samples were taken from these infants for testosterone assays when they were 1 to 3 months old. Parents (85 mothers, 2 fathers) completed an online questionnaire assessing their children’s autistic traits when the children were 18 to 30 months old (mean age = 22.39 months; *SD* = 3.44 months; range = 18.00–30.32 months). In the current sample, most of the children are of Caucasian descent (87.4 %); the rest are of mixed (11.5 %) or Indian/Pakistani/Bangladeshi descent (1.1 %). Parents provided informed consents for their own and their children’s participation. The study was approved by the Psychology Research Ethics Committee at Cambridge University.

#### Outcome variable

Autistic traits were measured by the Quantitative Checklist for Autism in Toddlers (Q-CHAT) [2], a 25-item parent-report questionnaire answered using a 5-point Likert scale. It has been validated in an unselected sample of children at the age of 18 to 24 months and in children with ASCs at the age of 19 to 63 months [2]. It has good test-retest reliability after 1 month (*r* = .82) [2]. Individual item scores are summed to obtain a Q-CHAT total score, ranging from 0 to 100. Higher scores indicate more autistic traits. Internal consistency (Cronbach’s α) based on all items of the Q-CHAT was .67 in the current sample of 87 children.

#### Predictor variable

Saliva was collected from the infant using a small-sized inert polymer swab at the age of 4 to 14 weeks (mean age = 7.82 weeks; *SD* = 1.96 weeks; range = 4.14–14.43 weeks). Testosterone exhibits a diurnal rhythm, with the highest concentrations in the morning and the lowest concentrations around midnight [19, 20], so saliva samples were collected between 8:30 am and 12 noon. There was no correlation between testosterone and sample collection time within this 3.5-h window in a subsample of 60 children for whom sampling time was recorded (*r* = .03). Samples were stored at −25 °C before being sent to Salimetrics (Cambridgeshire, England) for testosterone assays. Testosterone concentrations were measured in duplicate using enzyme immunoassays (assay sensitivity <1 pg/ml, intra-assay coefficient of variation = 5 %, inter-assay coefficient of variation = 10 %). One male infant produced insufficient saliva for hormonal assay. Therefore, data on testosterone concentrations were available for 39 boys and 47 girls.

#### Control variables

Birth weight, child’s age at saliva sampling and at Q-CHAT assessment, maternal and paternal age and education level, and number of siblings were included as control variables. Maternal and paternal education was rated on a 5-point scale from 1 (primary education only) to 5 (postgraduate degree).

### Results

**Fig. 1:**
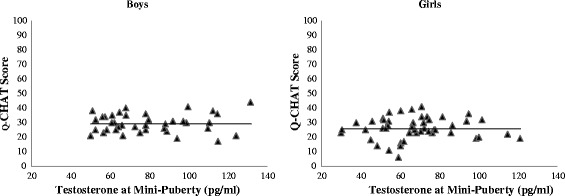
Scatter plot showing the relationship between Q-CHAT scores and testosterone at mini-puberty within each sex

There were no outliers and all variables had acceptable skewness. Boys and girls differed significantly in testosterone concentrations and in Q-CHAT scores but not in any other variable (see Table 1 for descriptive, independent sample *t* test and Cohen’s *d* statistics). There were no significant bivariate correlations between Q-CHAT scores and testosterone or any control variable in boys, girls, or the entire sample (see Table 2 for Pearson’s correlation statistics and Fig. 1 for a scatter plot showing the relationship between Q-CHAT scores and testosterone within each sex).

**Table 1:** Descriptive and inferential statistics for differences between boys and girls

	Boys (B)			Girls (G)			All			B vs. G		
	*n*	*M*	*SD*	*n*	*M*	*SD*	*n*	*M*	*SD*	*t*	*p*	*d* ^a^
Testosterone at mini-puberty (pg/ml)	39	79.68	22.56	47	67.98	20.19	86	73.29	21.97	2.54	.013	.55
Q-CHAT scores	40	28.83	6.89	47	25.74	7.23	87	27.62	7.24	2.06	.042	.43
Birth weight (kg)	40	3.39	.53	47	3.42	.43	87	3.41	.48	−.27	.790	−.06
Child’s age at saliva sampling (weeks)	40	7.62	1.67	47	8.00	2.19	87	7.82	1.96	−.90	.369	−.19
Child’s age at Q-CHAT assessment (months)	40	22.47	3.55	47	22.31	3.38	87	22.39	3.44	.22	.830	.05
Maternal age (years)	40	34.52	3.23	46	33.95	4.45	86	34.21	3.92	.67	.504	.14
Paternal age (years)	39	35.96	3.65	46	36.51	5.92	85	36.26	4.99	−.50	.620	−.11
Maternal education	40	4.65	.48	47	4.64	.53	87	4.64	.51	.11	.915	.02
Paternal education	40	4.50	.64	47	4.68	.52	87	4.60	.58	−1.46	.148	−.31
Number of siblings	40	.58	.87	47	.60	.74	87	.59	.80	−.12	.905	−.02

^a^Positive *d*s indicate higher values in boys than girls

*Note*. Maternal and paternal education was rated on a 5-point scale from 1 (primary education only) to 5 (postgraduate degree)

**Table 2:** Correlations of Q-CHAT scores to predictor and control variables

	Boys			Girls			All		
	*n*	*r*	*p*	*n*	*r*	*p*	*n*	*r*	*p*
Testosterone at mini-puberty	39	.07	.684	47	.03	.827	86	.11	.323
Birth weight	40	.03	.877	47	−.23	.121	87	−.11	.305
Child’s age at saliva sampling	40	.27	.084	47	−.06	.674	87	.04	.738
Child’s age at Q-CHAT assessment	40	−.14	.380	47	−.17	.243	87	−.15	.164
Maternal age	40	.15	.365	46	.08	.614	86	.12	.293
Paternal age	39	−.02	.885	46	.22	.136	85	.13	.243
Maternal education	40	−.03	.865	47	−.12	.418	87	−.08	.469
Paternal education	40	−.03	867	47	−.12	421	87	−.11	.328
Number of siblings	40	−.08	.632	47	.08	.599	87	.00	.980

Since the Q-CHAT was originally designed for children at the age of 18 to 24 months [2], the above analyses were repeated excluding children older than 24 months (12 boys, 12 girls). Highly comparable results were produced. There were no significant correlations between testosterone and Q-CHAT scores in boys (*r* = .09), girls (*r* = .03), or the entire sample (*r* = .13) for children ages 18 to 24 months.

### Conclusions

The present study investigated whether salivary testosterone measured at 1 to 3 months of age relates to autistic traits assessed by the Q-CHAT at 18 to 30 months of age. Consistent with prior studies [2, 8], the present study found that boys scored higher on the Q-CHAT than girls. Whereas prior studies reported no sex difference in salivary testosterone in infants at age 3 to 4 months [8, 18], the present study found that boys had higher concentrations of salivary testosterone than girls at age 1 to 3 months, during the peak of the early postnatal surge (mini-puberty). Despite detecting differences between boys and girls in both testosterone and autistic traits, the present study found no correlation between testosterone and autistic traits in boys, girls, or the entire sample. Although the current results cannot be directly compared with prior findings, due to the differences in the timing of testosterone measures, research findings thus far appear to suggest that testosterone during mini-puberty has little to no effects on autistic traits [8, 18].

In addition to augmenting evidence on testosterone and autistic traits, our findings highlight the potential of saliva sampling as an approach for studying the effects of early androgen exposure on neurobehavioural sexual differentiation. The present study detected a sex difference in salivary testosterone, which resembles prior findings showing sex differences in testosterone in blood [13, 14] and urine [21] samples collected during the peak of the postnatal surge. Because saliva can be obtained non-invasively and conveniently, its use may be particularly well-suited for further research investigating the effects of mini-puberty. Whilst mini-puberty may not contribute to autistic traits, it has been found that urinary testosterone and penile growth during mini-puberty predict gender-typed play behaviour in early childhood [21, 22]. Further research may relate salivary testosterone to other aspects of development that differ by gender.

The current results do not address the influences of testosterone prenatally. There is some evidence suggesting that testosterone exposure during foetal development may relate to later autistic traits [8–12]. By contrast, prior studies found no relationship between autistic traits and perinatal or postnatal androgen exposure, as measured in umbilical cord blood at birth or saliva after the peak of mini-puberty [8, 18, 23, 24]. Moreover, the present study found no relationship between testosterone at the peak of mini-puberty and autistic traits. Thus, research evidence thus far appears to suggest that early postnatal testosterone does not contribute to autistic traits.

Limitations of the present study include reliance on a small sample, which could reduce statistical power, and the largely Caucasian and highly educated participants (see Table 1), which could reduce generalisability of the findings. Further research with larger and more diverse samples would be of interest.

In conclusion, the present study expands on prior research, suggesting that there is no relationship between testosterone exposure during mini-puberty and autistic traits. This does not preclude effects of mini-puberty on other behaviours, however. Indeed, testosterone during mini-puberty has been found to predict gender-typed play behaviour. Future studies might use early postnatal saliva samples to evaluate the effects of mini-puberty on other traits that show gender differences.

## Abbreviations

ASC: autism spectrum condition
BITSEA: Brief Infant–Toddler Social and Emotional Assessment
EMB: extreme male brain
Q-CHAT: Quantitative Checklist for Autism in Toddlers
